# Supplementary figures and images for: Single-cell transcriptomics of the naked mole-rat reveals unexpected features of mammalian immunity
Source: PLoS Biol. 2019 Nov 21;17(11):e3000528. doi: 10.1371/journal.pbio.3000528 (PMC6894886; doi:10.1371/journal.pbio.3000528)

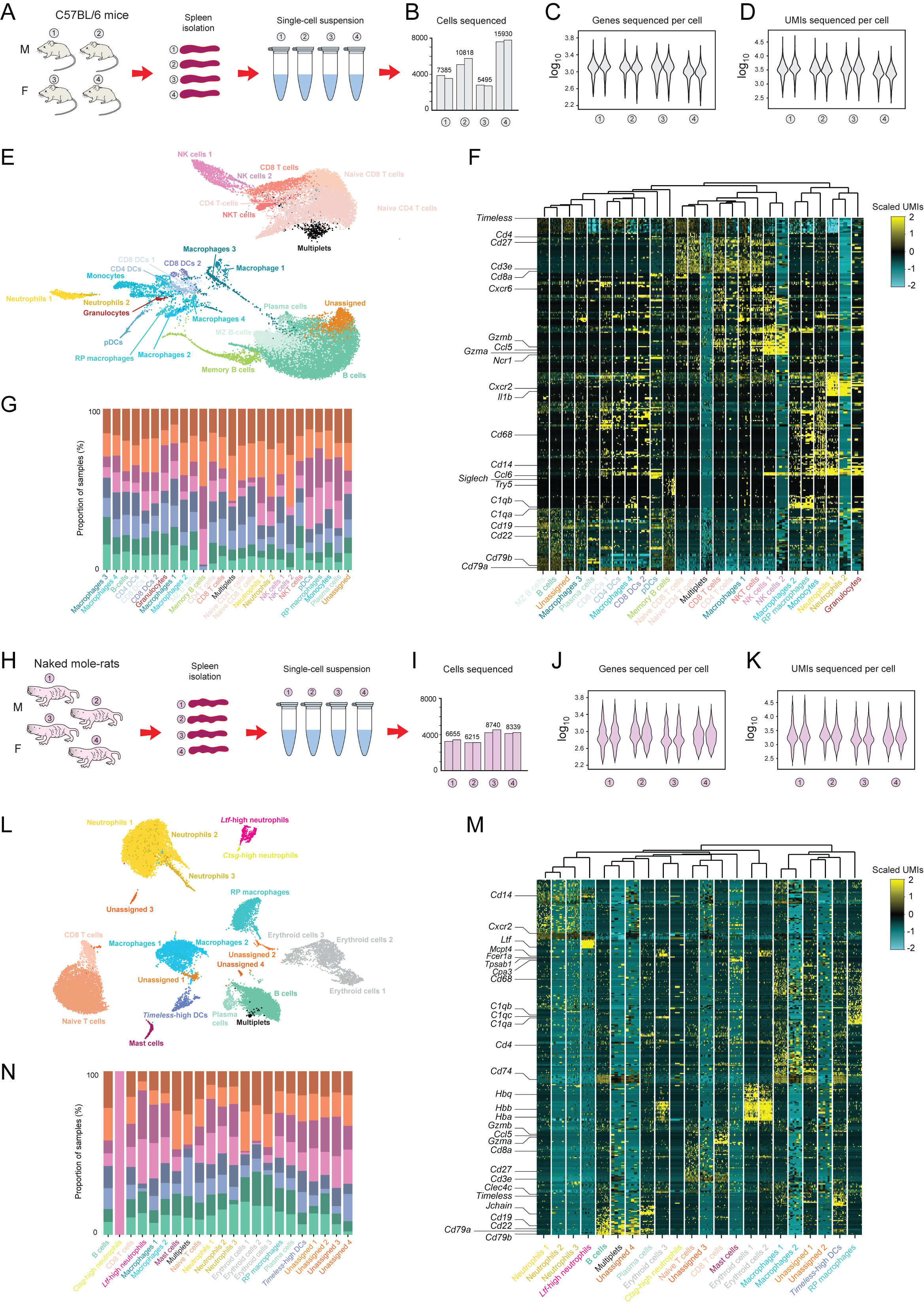

Supplement: S1 Fig — (A) Schematic view showing the workflow in which single-cell suspensions are derived from four (two males [M], two females [F]) C57BL/6 mouse spleens. (B) Bar chart showing the number of cells sequenced in duplicate from each of the four mouse spleens (see S43 Table for underlying data). (C) Violin plot showing the number of genes sequenced per cell from each of the four mouse spleens (see S1 Table for underlying data). (D) Violin plot showing the number of UMIs sequenced per cell from each of the four mouse spleens (see S1 Table for underlying data). (E) UMAP projection of the four-mouse spleen scRNA-seq data, for which each point is a cell color-coded by its converged-cluster assignment and annotated cell type. (F) Gene-by-cell expression-level heatmap of the four-mouse spleen scRNA-seq data, in which selected marker genes are listed to the left and cells are faceted by their converged cluster assignment. (G) Stacked bar chart showing the proportion (%) of cells from each of the four mouse duplicate spleen samples assigned to each of the converged clusters (see S1 Table for underlying data). Samples are color-coded and duplicates are shade-coded. (H) Schematic view showing the workflow in which single-cell suspensions are derived from four (two males [M], two females [F]) NM-R spleens. (I) Bar chart showing the number of cells sequenced in duplicate from each of the four NM-R spleens (see S43 Table for underlying data). (J) Violin plot showing the number of genes sequenced per cell from each of the four NM-R spleens (see S2 Table for underlying data). (K) Violin plot showing the number of UMIs sequenced per cell from each of the four NM-R spleens (see S2 Table for underlying data). (L) UMAP projection of the four NM-R spleen scRNA-seq data, for which each point is a cell color-coded by its converged-cluster assignment and annotated cell type. (M) Gene-by-cell expression-level heatmap of the four NM-R spleen scRNA-seq data, in which selected marker genes are listed [file pbio.3000528.s001.tif]

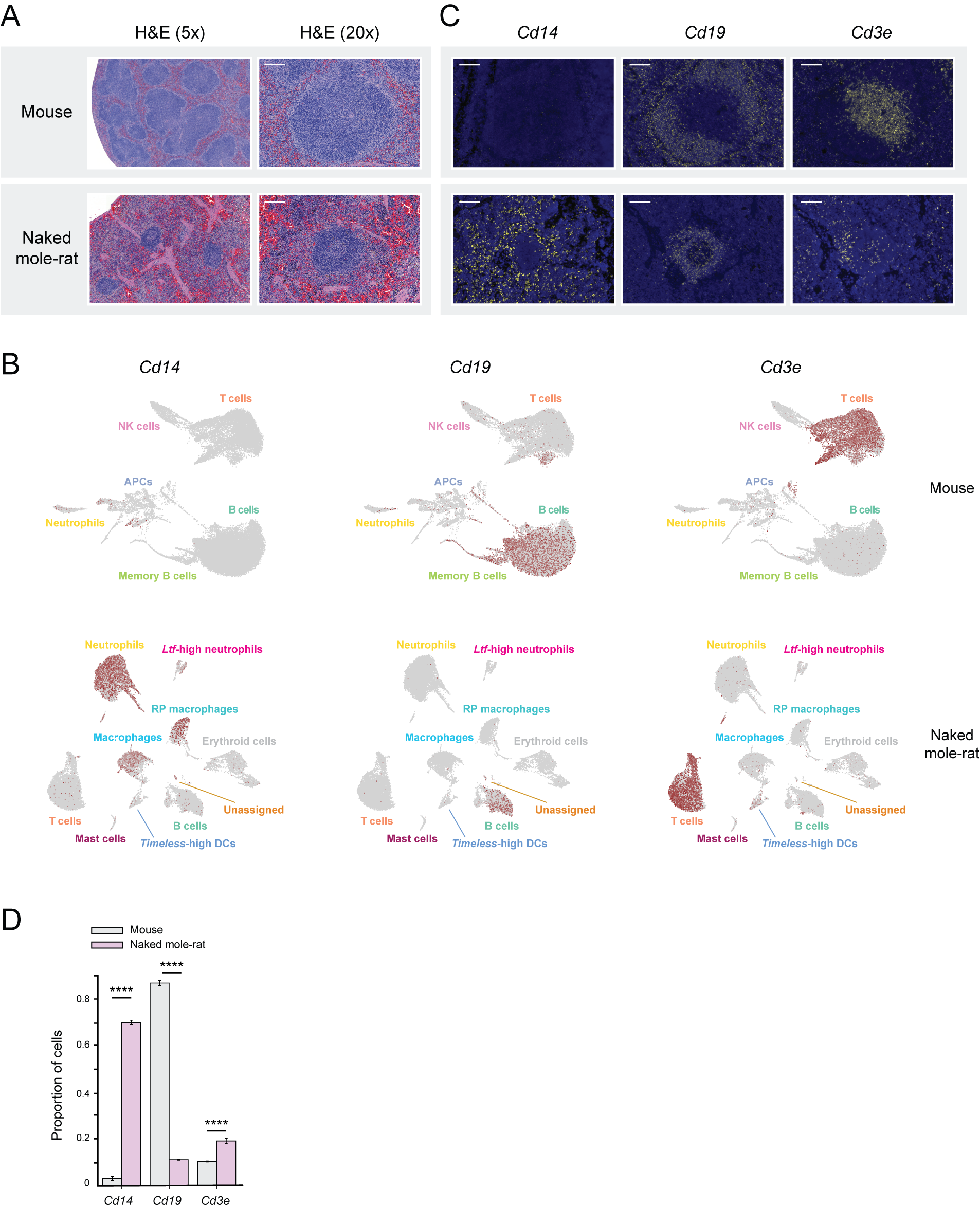

Supplement: S2 Fig — (A) Representative images of HE-stained consecutive sections of mouse (upper panel) and NM-R (lower panel) spleens shown at 5× (left panels) and 20× magnifications (right panels), scale bar = 100 μm. Mouse and NM-R spleens show major differences in splenic microanatomy, with the NM-R having a comparatively reduced white-pulp compartment and a larger red-pulp compartment with a greater number of fibromuscular trabeculae that connect to the capsule and provide structural support and contractility to the spleen. Within the white pulp, the marginal zone and follicles of the NM-R (which comprise B cells) are readily identifiable. By contrast, the PALS (which comprises the T cell–rich compartment in other species) is less prominent in the NM-R than in the mouse. (B) UMAP projections of the mouse (upper panels) and NM-R (lower panels) spleen scRNA-seq data color-coded by the expression levels of myeloid and lymphoid lineage marker genes: Cd14 (myeloid marker), Cd19 (B cell marker, lymphoid), and Cd3e (T cell marker, lymphoid). (C) Representative consecutive sections of mouse (upper panel) and NM-R (lower panel) spleens showing results from ISH staining for Cd14, Cd19, and Cd3e. Positive expression of the marker genes was visualized with a TRITC filter (yellow) and nuclei visualized using DAPI staining (blue). Magnification, 20×. Scale bar = 100 μm. Expression of the housekeeping gene Rpl13a and the bacterial-specific dapB gene were included as positive and negative controls, respectively (Materials and methods). (D) Quantification of the ISH staining of Cd14, Cd19, and Cd3e in mouse (gray bars) and NM-R (pink bars). Bars represent the proportion of positive staining cells (n = 4 biological replicates) and error bars represent the uncertainty associated with the number of puncta used as a cutoff for defining a cell as positive for a marker gene (Materials and methods and S5 Table for underlying data). Asterisks mark statistically significant (adjusted p < 0.05) differences [file pbio.3000528.s002.tif]

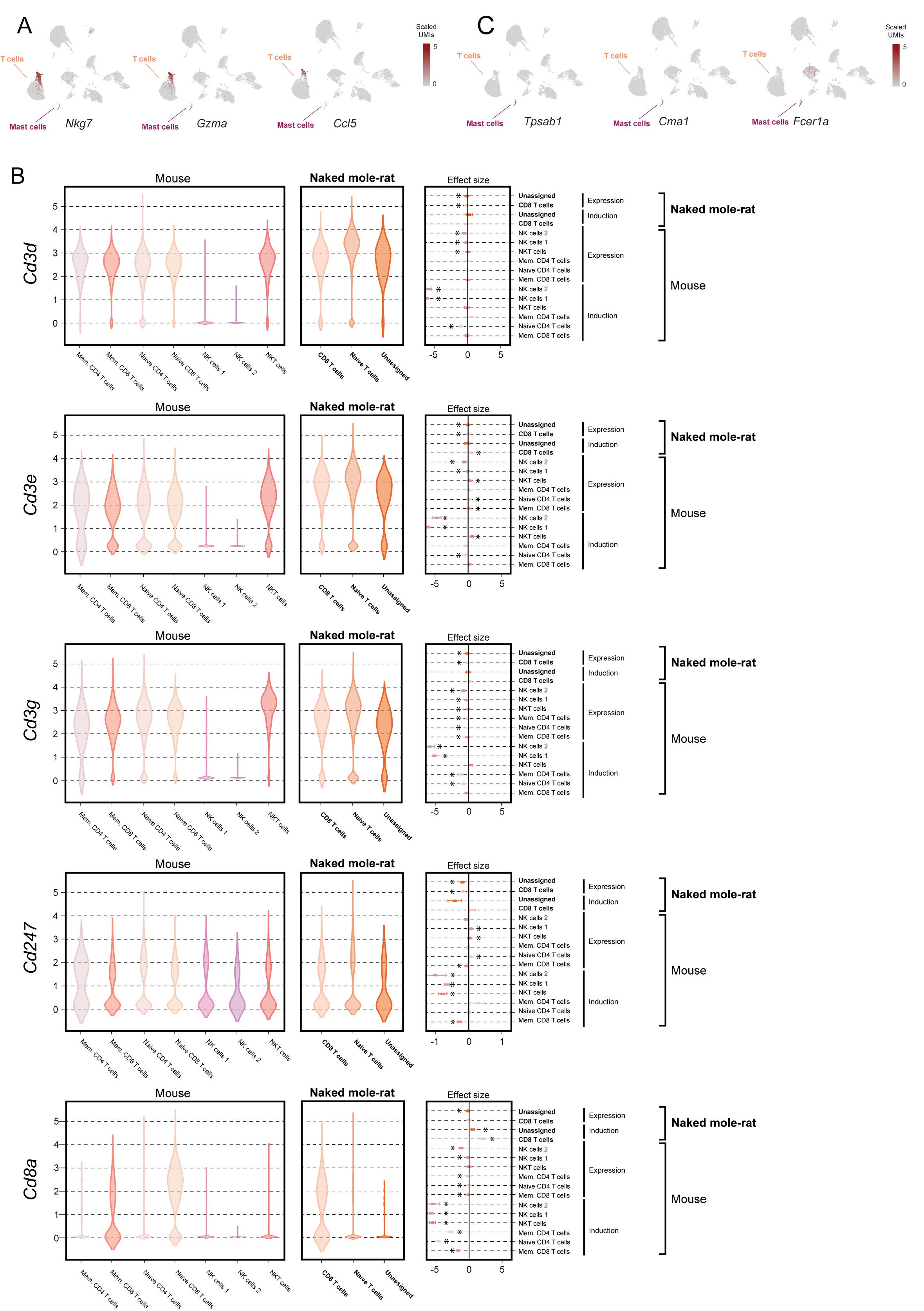

Supplement: S3 Fig — (A) UMAP projections of the four NM-R spleens scRNA-seq data color-coded by the expression levels of three canonical NK cell marker genes: Nkg7, Gzma, and Ccl5. (B) Violin plots showing the expression levels of the CD3 subunit genes: Cd3d, Cd3e, Cd3g, Cd247, and of Cd8a in the mouse T-cell and NK cell converged clusters (left panel) and NM-R T-cell converged clusters (center panel). The side panels show effect sizes for expression-level changes and expression-induction changes, where in the mouse, the naïve CD8 T cells are used as baseline and in the NM-R, the naïve T cells are used as baseline (see S6 Table for underlying data). Asterisks mark adjusted p < 0.05. (C) UMAP projections of the four NM-R spleens scRNA-seq data color-coded by the expression levels of three mast cells marker genes: Tpsab1, Cma1, and Fcer1a. CD3, cluster of differentiation 3; Mem, memory; NK, natural killer; NKT, natural killer T; NM-R, naked mole-rat; scRNA-seq, single-cell RNA-sequencing; UMAP, uniform manifold approximation and projection. (TIF) [file pbio.3000528.s003.tif]

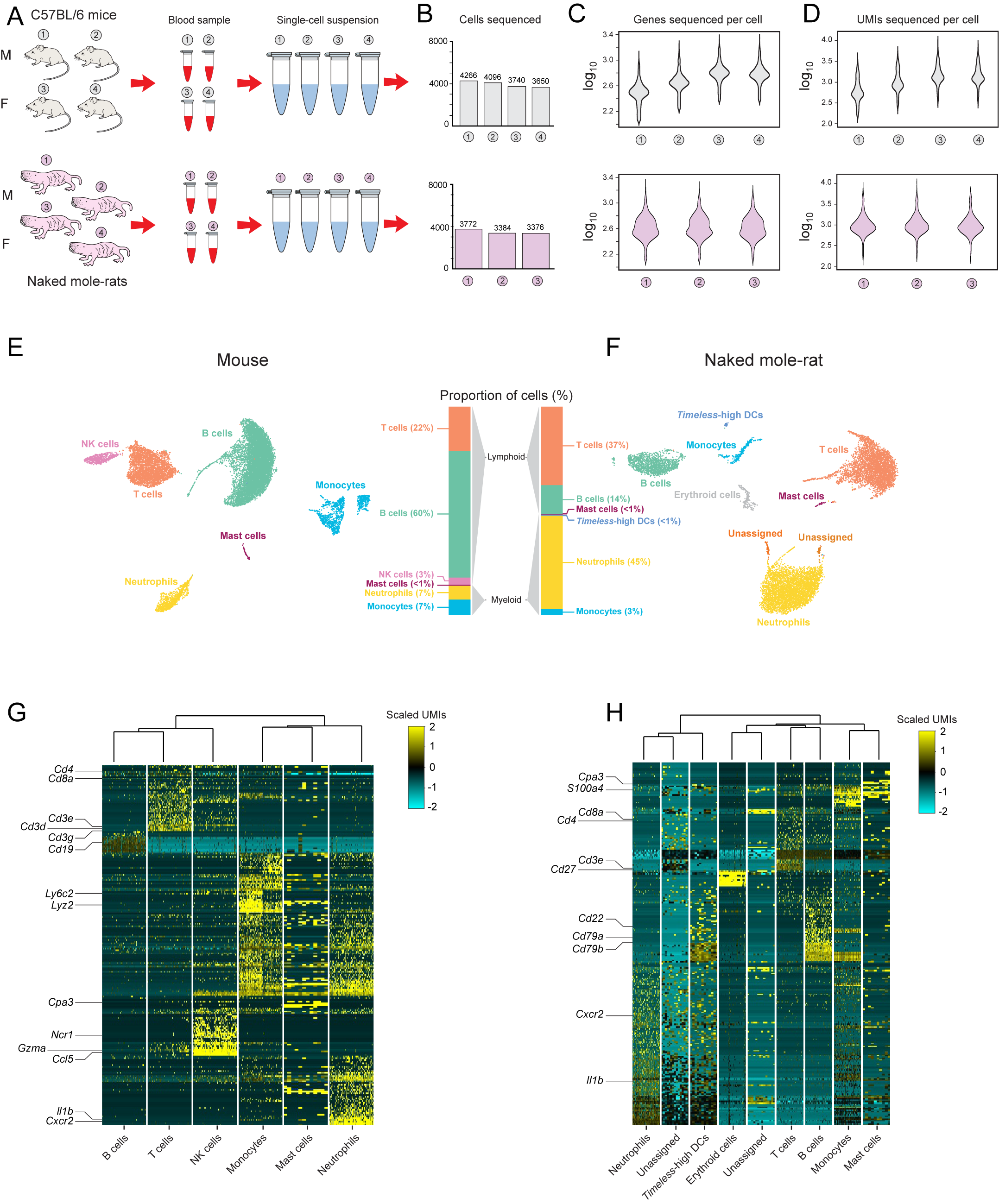

Supplement: S4 Fig — (A) Schematic view showing the workflow in which single-cell suspensions are derived from circulating immune cells of four (two males [M], two females [F]) mice (upper panel, gray) and four NM-Rs (two males [M], two females [F]) (lower panel, pink). (B) Bar charts showing the number of cells sequenced from each of the four mice (upper panel, gray) and three NM-Rs (one sample was lost due to a microfluidic failure during the emulsion generation; lower panel, pink) (see S43 Table for underlying data). (C) Violin plots showing the number of genes sequenced per cell from each of the four mice (upper panel, gray; see S7 Table for underlying data) and three NM-Rs (lower panel, pink; see S8 Table for underlying data). (D) Violin plots showing the number of UMIs sequenced per cell from each of the four mice (upper panel, gray; see S7 Table for underlying data) and three NM-Rs (lower panel, pink; see S8 Table for underlying data). UMAP projections of the four mouse (E) and three NM-R (F) circulating immune scRNA-seq datasets. Each point is a cell color-coded by its first iteration cluster assignment and annotated cell type. The proportions of each cell type are shown in the central bar chart. Gene-by-cell expression-level heatmaps of the four mouse (G) and three NM-R (H) circulating immune scRNA-seq datasets. Selected marker genes are listed to the left and cells are faceted by their first iteration cluster assignment. DC, dendritic cell; NK, natural killer; NM-R, naked mole-rat; scRNA-seq, single-cell RNA-sequencing; UMAP, uniform manifold approximation and projection; UMI, unique molecular identifier. (TIF) [file pbio.3000528.s004.tif]

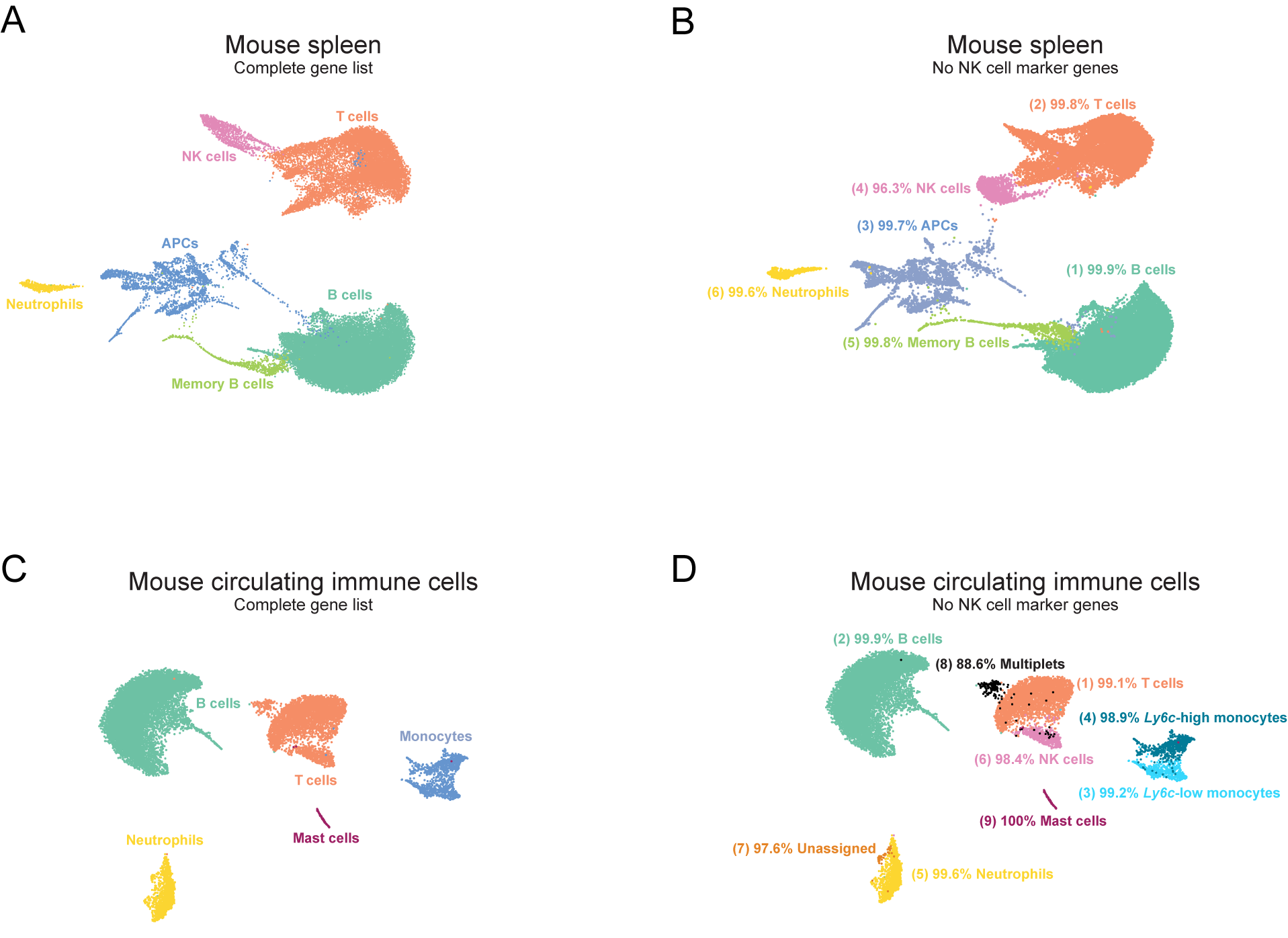

Supplement: S5 Fig — One possibility for the absence of NK cells in the NM-R may be that, due to incomplete annotation of NK cell marker genes in the NM-R, genome reads generated from these marker genes would be filtered as a result of not mapping back to the annotated genome. To test this possibility, we simulated a lack of NK cell marker genes in the mouse by selectively eliminating the mouse NK cell marker genes (51 and 43 genes in the spleen and circulating immune cells; S3 and S9 Tables, respectively) and subsequently re-clustered the mouse data (done both for the spleen data as well as for the circulating immune cells). (A) UMAP projection of the four mouse spleen scRNA-seq data color-coded by the first iteration clusters achieved using the full set of genes. (B) UMAP projection of the four mouse spleen scRNA-seq data color-coded by the first iteration clusters achieved using the set of genes without the mouse spleen NK cell marker genes. Clusters are labeled by their indices and by the percentage of their cells that map to the cluster/cell type they correspond to in (A). (C) UMAP projection of the four mouse circulating immune scRNA-seq data color-coded by the first iteration clusters achieved using the full set of genes. (D) UMAP projection of the four mouse circulating immune scRNA-seq data color-coded by the converged clusters achieved using the set of genes without the mouse circulating NK cell marker genes. Clusters are labeled by their indices and by the percentage of their cells that map to the cluster/cell type they correspond to in (C). APC, antigen-presenting cell; NK, natural killer; NM-R, naked mole-rat; scRNA-seq, single-cell RNA-sequencing; UMAP, uniform manifold approximation and projection. (TIF) [file pbio.3000528.s005.tif]

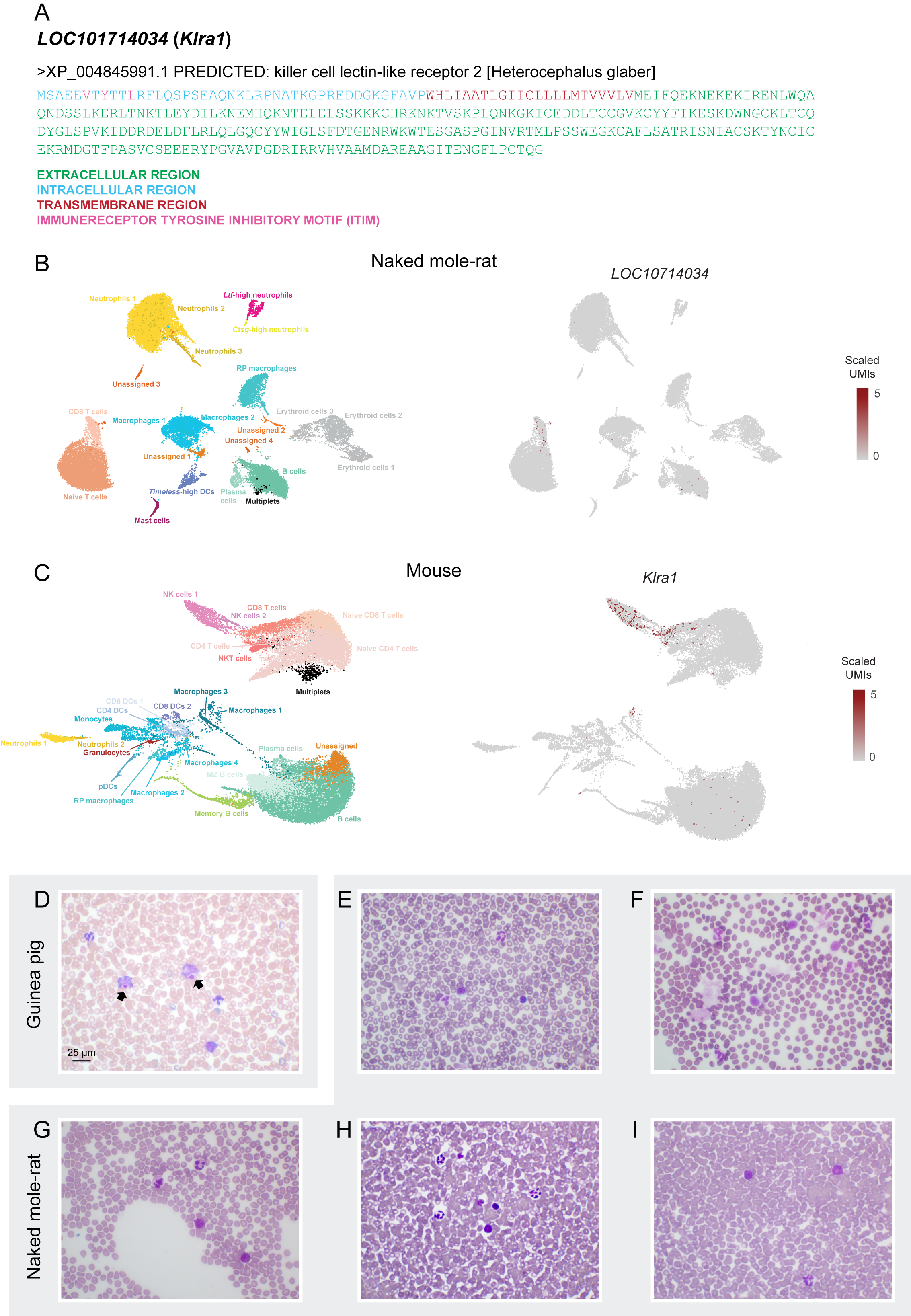

Supplement: S6 Fig — (A) The amino acid sequence of the NM-R Ly49 gene (LOC101714034; a mouse Klra1 ortholog) color-coded by its predicted functional domains: extracellular region (green), intracellular region (blue), and transmembrane region (red). The immunoreceptor tyrosine inhibitory motif (ITIM) is shown in pink. (B) UMAP projections of the NM-R spleen scRNA-seq data color-coded by clusters (left) and by the expression levels of LOC101714034 (right), showing its low expression levels in CD8 T cells. (C) UMAP projections of the mouse spleen scRNA-seq data color-coded by clusters (left) and by the expression levels of Klra1 (right), showing its high expression levels in CD8 T cells and NK cells. (D) Wrights-Giemsa stained peripheral blood smear (magnification = 60×; scale bar = 25 μm) from a young female guinea pig, with arrows marking Foa-Kurloff cells characterized by a large cuniform cytoplasmic inclusion body, along with two polymorphonuclear neutrophils (top left and center) and a lymphocyte (bottom). (E-I) Wrights-Giemsa stained peripheral blood smears (magnification = 60×; scale bar = 25 μm) from five young NM-Rs ([E] 2-month-old male, [F] 7-month-old male, [G] 15-month-old female, [H] 11-month-old male, [I] 7-month-old female) showing mainly polymorphonuclear neutrophils and clumped platelets, along with a few lymphocytes, monocytes, and eosinophils with granular cytoplasm, where none of these leukocytes show an overt Foa-Kurloff cell morphology. CD8, cluster of differentiation 8; DC, dendritic cell; NK, natural killer; NKT, natural killer T; NM-R, naked mole-rat; pDC, plasmacytoid dendritic cell; RP, red pulp; scRNA-seq, single-cell RNA-sequencing; UMAP, uniform manifold approximation and projection. (TIF) [file pbio.3000528.s006.tif]

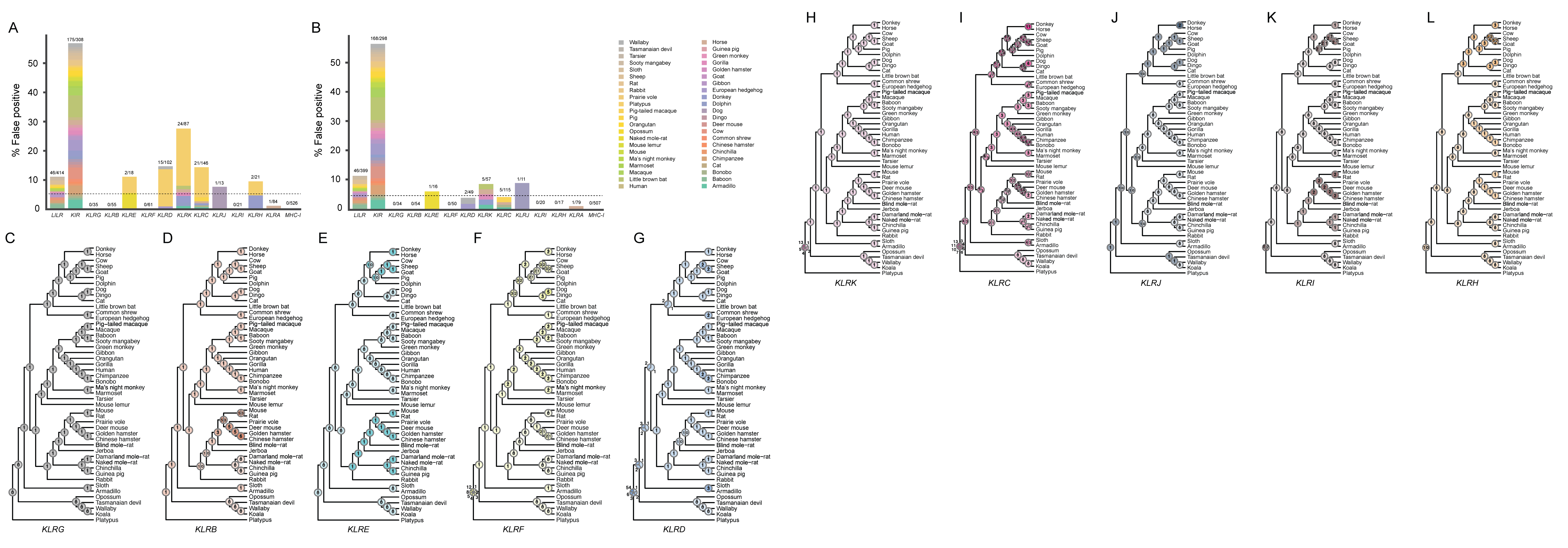

Supplement: S7 Fig — (A) Stacked bar chart showing the benchmarked percentage of false positive putative NK cell receptor and MHC-I pseudogenes (Materials and methods and S12 Table for underlying data) for each gene family (bars), color-coded by the genome of the search species. Labeled on top of each bar is (number of false positives)/(number of true + false positives). Horizontal black dashed line marks the 5% false positive rate. While the high false positive rate in the LILR and KIR gene families is driven by many of the search genomes, for the remaining gene families the main drivers of the false positive rate are the platypus and donkey search genomes (orange and purple shades, respectively). (B) A similar stacked bar chart as (A) with the platypus and donkey search genomes removed, showing that in the gene families other than LILR and KIR, all gene families except for KLRK have either a single false positive or a false positive rate <5%. (C-L) Phylogenies of the 48 mammalian genomes; each corresponds to one of the NK cell receptor gene families within the NKC (except for KLRA): KLRG (C), KLRB (D), KLRE (E), KLRF (F), KLRD (G), KLRK (H), KLRC (I), KLRJ (J), KLRI (K), and KLRH (L). Color shades correspond to the numbers of genes. Because the ancestral reconstruction is not deterministic, ancestral counts are represented as pie charts corresponding to the probability of each count. KIR, killer cell immunoglobulin-like receptor; KLR, killer cell lectin-like receptor; LILR, leukocyte immunoglobulin-like receptor; MHC-I, major histocompatibility complex class I; NK, natural killer; NKC, natural killer cell receptor complex. (TIF) [file pbio.3000528.s007.tif]

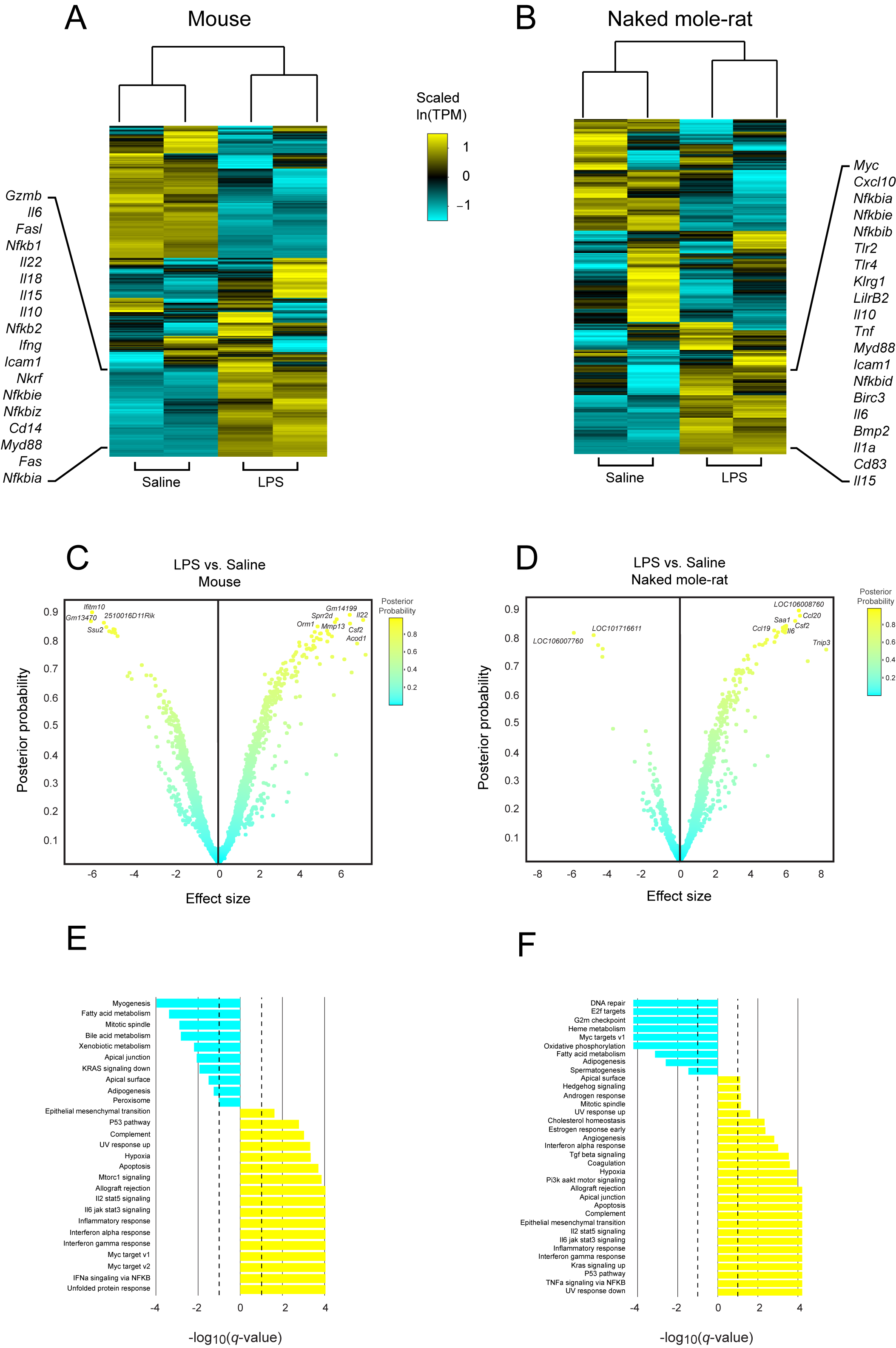

Supplement: S8 Fig — (A) Genes-by-samples heatmap showing the scaled ln(TPM) expression levels of genes in spleens from the two saline control and two LPS-challenged mice. Selected LPS up-regulated genes are shown to the left. (B) Genes-by-samples heatmap showing the scaled ln(TPM) expression levels in spleens from the two saline control and two LPS-challenged NM-Rs. Selected LPS up-regulated genes are shown to the right. (C) Volcano plot showing the posterior probability of the estimated effect size (ln(LPS/saline) ln(TPM) fold-change) being different from zero (y-axis) versus the estimated effect size (x-axis) in the mouse data. Each point is a gene, and the color code follows the posterior probability gradient. (D) Volcano plot showing the posterior probability of the estimated effect size (ln(LPS/saline) ln(TPM) fold-change) being different from zero (y-axis) versus the estimated effect size (x-axis) in the NM-R data. Each point is a gene, and the color code follows the posterior probability gradient. (E) Bar chart showing the Hallmark gene sets enriched in genes with strong expression changes following LPS challenge in mice (see S16 Table for underlying data). The x-axis reports the log10-adjusted p-value (q-value) of the GSEA, signed by the direction of the effect (up- and down-regulation in LPS relative to saline are represented as follows: positive is yellow and negative is cyan, respectively). Vertical dashed lines represent adjusted p = 0.1. (F) Bar chart showing the Hallmark gene sets enriched in genes with strong expression changes following LPS challenge in NM-Rs (see S17 Table for underlying data). The x-axis reports the log10-adjusted p-value (q-value) of the GSEA, signed by the direction of the effect (up- and down-regulation in LPS relative to saline are represented as follows: positive is yellow and negative is cyan, respectively). Vertical dashed lines represent adjusted p = 0.1. GSEA, gene set enrichment analysis; LPS, lipopolysaccharide; NM-R, naked mole-rat; TPM, t [file pbio.3000528.s008.tif]

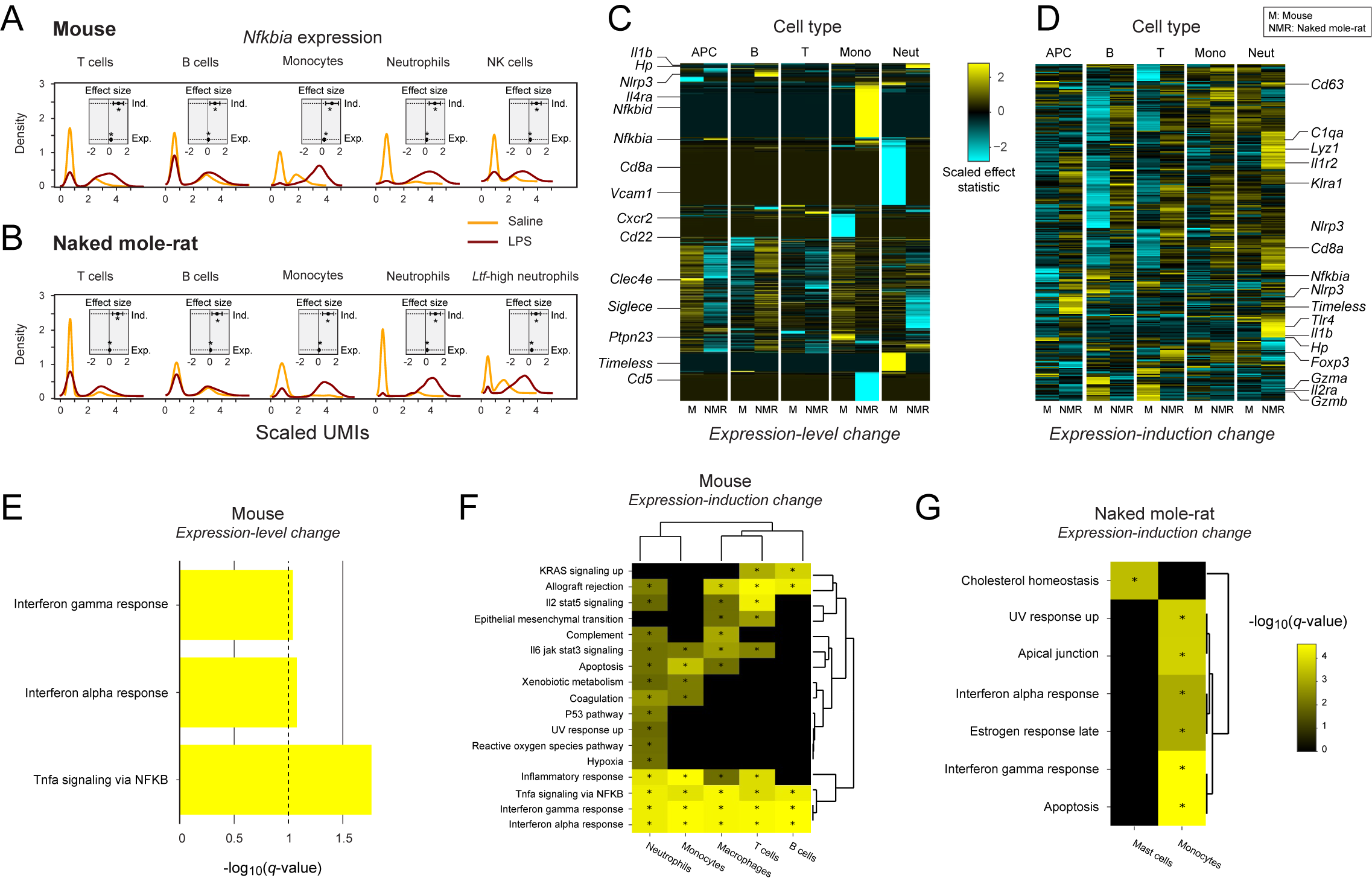

Supplement: S9 Fig — Marked transcriptional responses were evident following LPS challenge for each cell type compared between the LPS-challenged and saline control animals in each of the species. As previously described by Shalek and colleagues [52], these LPS-responsive transcriptional changes were driven by expression-level change (a change in the level of the expression among the cells that express a given gene in both conditions) and/or expression-induction change (a change in the proportion of cells expressing a certain gene in both conditions), illustrated here by the transcriptional changes of Nfkbia. (A) Selected density plots showing the expression distribution of Nfkbia from saline control (orange) and LPS-challenged (red) mice across the cells of five representative cell types. Insets show the estimated LPS-challenge expression-level change (Exp.) and expression-induction change (Ind.) effect sizes (LPS relative to saline) (asterisks mark adjusted p < 0.05). (B) Selected density plots showing the expression distribution of Nfkbia from saline control (orange) and LPS-challenged (red) NM-Rs across the cells of five representative cell types. Insets show the estimated LPS-challenge expression-level change (Exp.) and expression-induction change (Ind.) effect sizes (LPS relative to saline) (asterisks mark adjusted p < 0.05). (C) Heatmap showing the estimated LPS-challenge effect statistics for expression-level change. Selected marker genes are shown to the left. (D) Heatmap showing the estimated LPS-challenge effect statistics for expression-induction change. Selected marker genes are shown to the right. (E) Bar chart showing the GSEAs of the intraspecies LPS-challenge effect on expression-level change in mouse NK cells (see S26 Table for underlying data). The x-axis is the GSEA log10-adjusted p-values (q-values), signed and color-coded by the direction of the effect (up- and down-regulation in LPS-challenge relative to saline control are represented as yellow and cyan, respectiv [file pbio.3000528.s009.tif]

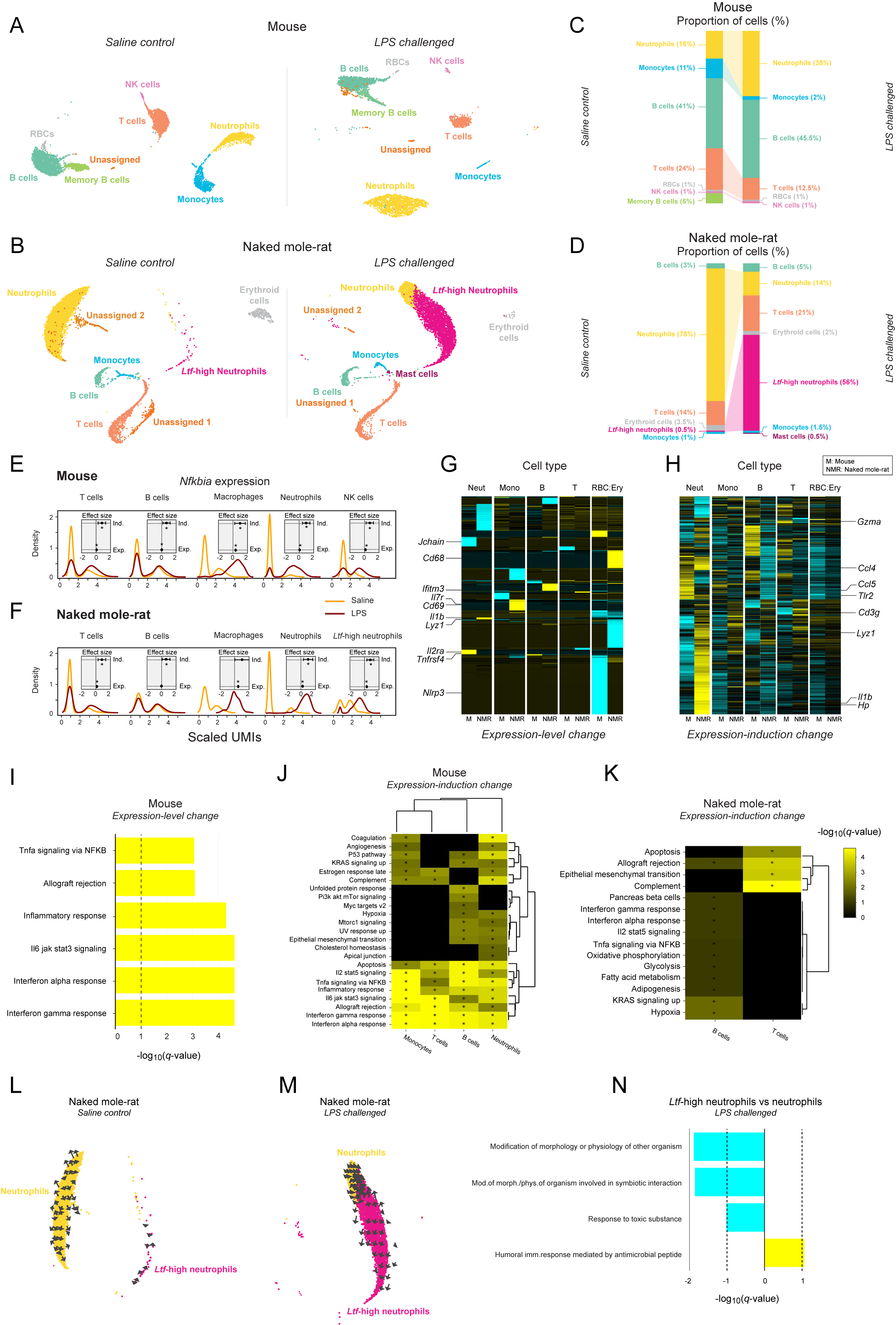

Supplement: S10 Fig — UMAP projections showing the clusters of the circulating immune cells from the two saline control (left panel) and two LPS-challenged (right panel) mice (A) and NM-Rs (B). Each point is a cell color-coded by its cluster assignment and annotated cell type. Stacked bar charts showing the proportions (%) of each cell type in the saline control (top) and LPS-challenged (bottom) mouse (C) and NM-R (D) circulating immune cells. Similar to the spleen saline control and LPS challenge data (S9 Fig), marked transcriptional responses were evident following LPS challenge for each cell type compared between the LPS-challenged and saline control animals in each of the species. And these changes were again driven by expression-level changes and/or expression-induction changes, illustrated here by the transcriptional changes of Nfkbia. (E) Selected density plots showing the expression distribution of Nfkbia from saline control (orange) and LPS-challenged (red) mice across the cells of five representative cell types. Insets show the estimated LPS-challenge expression-level change (Exp.) and expression-induction change (Ind.) effect sizes (LPS relative to saline) (asterisks mark adjusted p < 0.05). (F) Selected density plots showing the expression distribution of Nfkbia from saline control (orange) and LPS-challenged (red) NM-Rs across the cells of five representative cell types. Insets show the estimated LPS-challenge expression-level change (Exp.) and expression-induction change (Ind.) effect sizes (LPS relative to saline) (asterisks mark adjusted q < 0.05). (G) Heatmap showing the estimated LPS-challenge effect statistics for expression-level change. Selected marker genes are shown to the left. (H) Heatmap showing the estimated LPS-challenge effect statistics for expression-induction change. Selected marker genes are shown to the right. (I) Bar chart showing the GSEAs of the intraspecies LPS-challenge effect on expression-level change in mouse NK cells (see S39 Table for underlyin [file pbio.3000528.s010.tif]

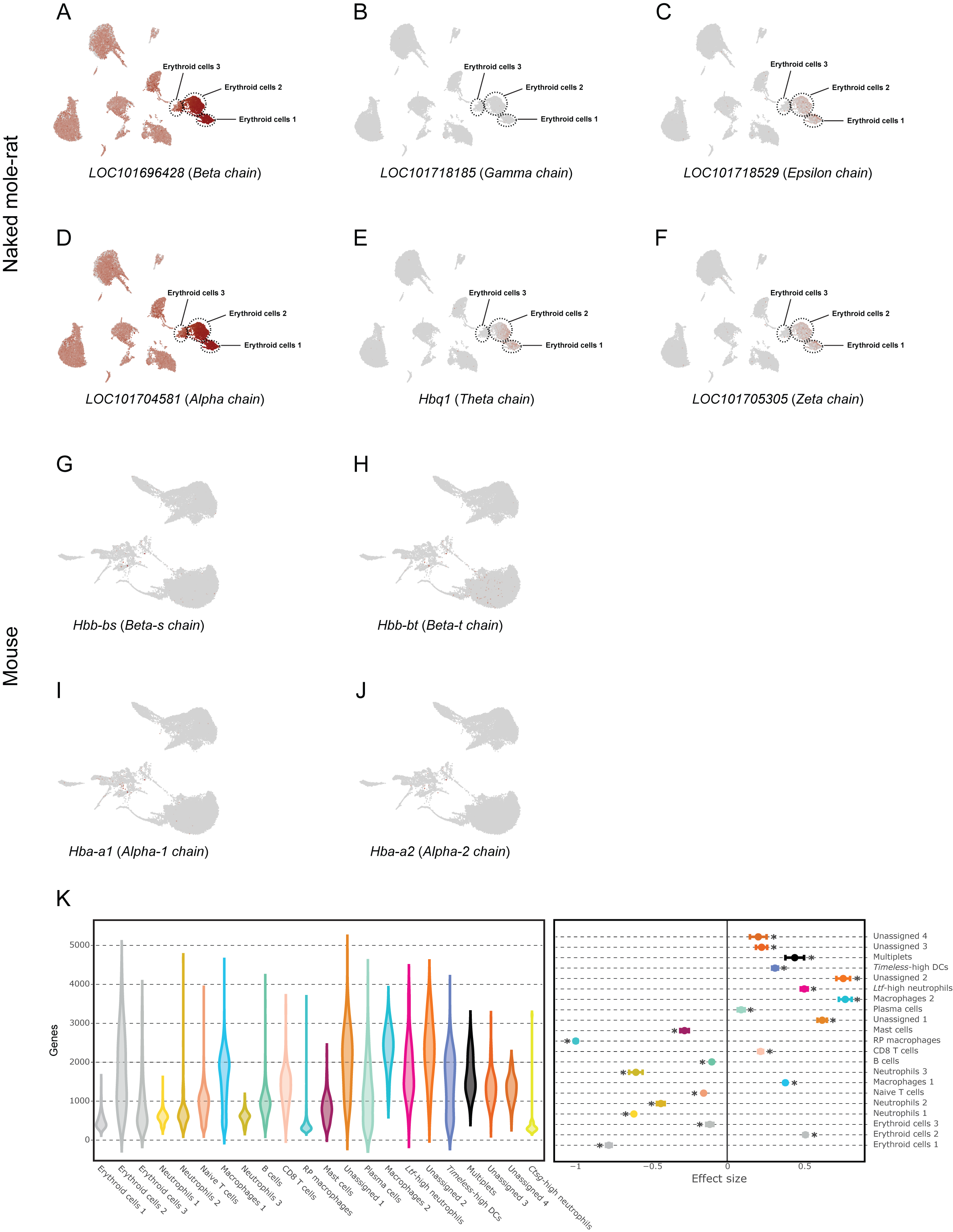

Supplement: S11 Fig — UMAP projections of the clusters of the NM-R and mouse spleen datasets color-coded by the expression levels of (A) NM-R beta hemoglobin, (B) NM-R gamma hemoglobin, (C) NM-R epsilon hemoglobin, (D) NM-R alpha hemoglobin, (E) NM-R theta hemoglobin, (F) NM-R zeta hemoglobin, (G) mouse beta-s hemoglobin, (H) mouse beta-t hemoglobin, (I) mouse alpha-1 hemoglobin, and (J) mouse alpha-2 hemoglobin. (K) Violin plot showing the numbers of genes expressed in each of the NM-R spleen converged clusters (right panel) and a plot showing the effect sizes comparing the number of genes in each of the NM-R converged clusters to the mean across all converged clusters (right panel; see S45 Table for the underlying data). Asterisks mark adjusted p < 0.05. DC, dendritic cell; NM-R, naked mole-rat; RP, red pulp; scRNA-seq, single-cell RNA-sequencing; UMAP, uniform manifold approximation and projection. (TIF) [file pbio.3000528.s011.tif]

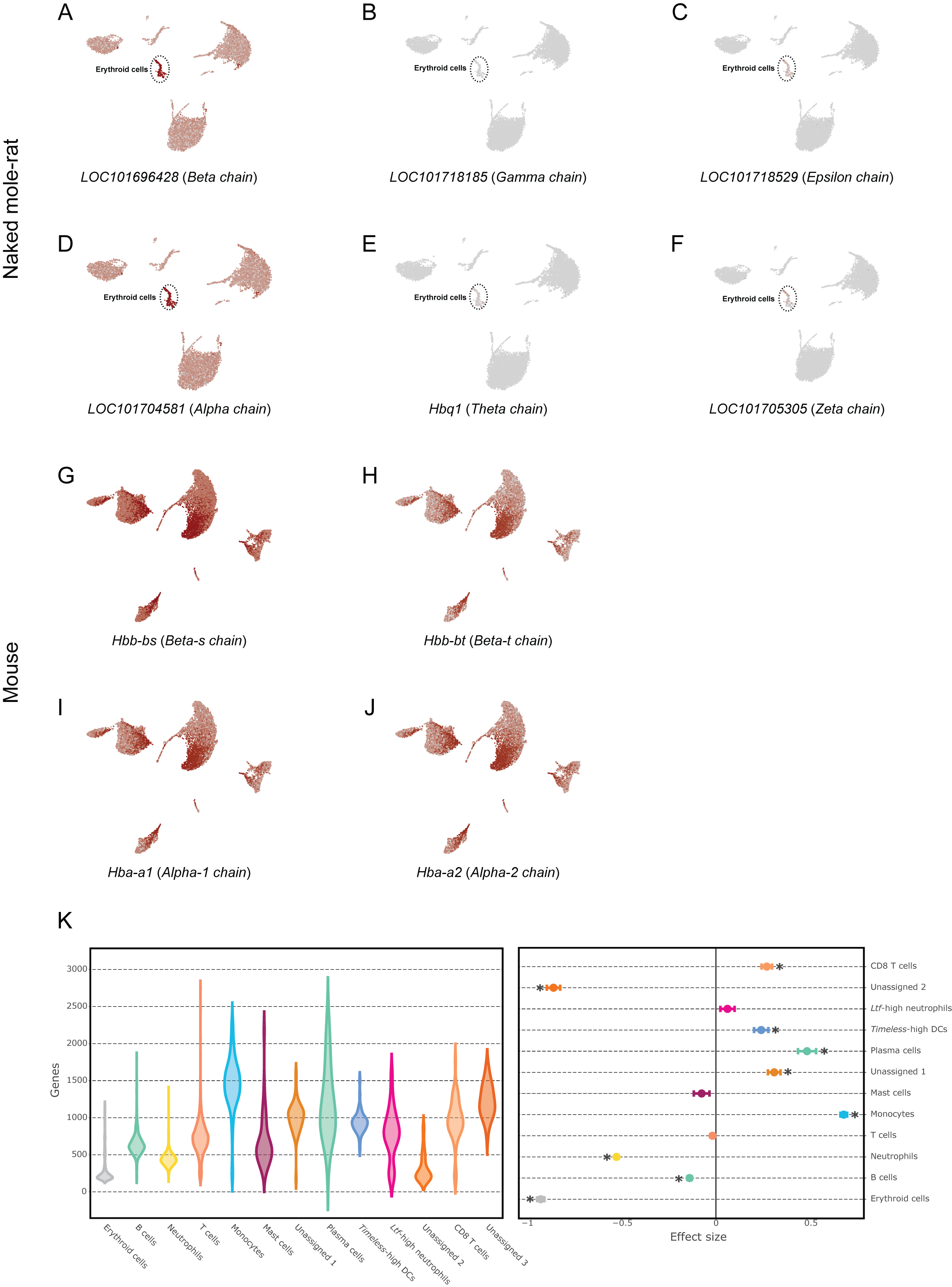

Supplement: S12 Fig — UMAP projections of the clusters of the NM-R and mouse circulating immune cell datasets color-coded by the expression levels of (A) NM-R beta hemoglobin, (B) NM-R gamma hemoglobin, (C) NM-R epsilon hemoglobin, (D) NM-R alpha hemoglobin, (E) NM-R theta hemoglobin, (F) NM-R zeta hemoglobin, (G) mouse beta-s hemoglobin, (H) mouse beta-t hemoglobin, (I) mouse alpha-1 hemoglobin, and (J) mouse alpha-2 hemoglobin. (K) Violin plot showing the numbers of genes expressed in each of the converged clusters of the NM-R circulating immune cells data (right panel) and a plot showing the effect sizes comparing the number of genes in each of the NM-R converged clusters to the mean across all converged clusters (right panel; see S46 Table for the underlying data). Asterisks mark adjusted p < 0.05. DC, dendritic cell; NM-R, naked mole-rat; scRNA-seq, single-cell RNA-sequencing; UMAP, uniform manifold approximation and projection. (TIF) [file pbio.3000528.s012.tif]
