# Supplementary material for: Single-cell transcriptomics of the naked mole-rat reveals unexpected features of mammalian immunity
Source: PLoS Biol. 2019 Nov 21;17(11):e3000528. doi: 10.1371/journal.pbio.3000528 (PMC6894886; doi:10.1371/journal.pbio.3000528)
Supplement: Attachment — Submitted filename: response.docx [file pbio.3000528.s065.docx]

**We thank the reviewers and editor for their constructive and generally favorable comments and have addressed all the concerns as point-by-point responses below.**

**Reviewer #1 (Ewan St. John Smith, signed review):**

The study from Hilton et al., is the first to make a comprehensive study of the immune system of the naked mole-rat. Considering the unusual biology of the naked mole-rat, e.g. extreme longevity and general resistance to the processes associated with ageing (as demonstrated through its resistance to cancer and neurodegeneration that are both associated with ageing) it is extremely important to understand more about naked mole-rat immune system, a system that generally deteriorates with ageing.

The authors use unbiased single cell RNA-sequencing of the spleens from mice and naked mole-rats to determine the comparative constituents of the immune systems of these two species. Data acquired from analysis of mouse splenic immune cells are consistent with what others have shown (e.g. a high proportion of lymphoid lineage B and T cells and low proportion of myeloid cells), whereas splenic immune cells of the naked mole-rat form a strikingly different composition, i.e. more myeloid than lymphoid lineage cells (including two subgroups of neutrophils) and an apparent absence of natural killer cells.

Details on the absence of natural killer cells from the spleen are provided and further single cell RNA-sequencing is conducted on circulating immune cells, which, along with efforts to simulate the possible poor annotation of natural killer cell marker genes in the naked mole-rat genome, confirms that naked mole-rats really do appear to lack a natural killer cell population. This finding correlates with a lack of expansion of genes like Ly49 that are associated with natural killer cell function. A broader comparison of 45 mammalian species places the naked mole-rat data in a wider context, demonstrating that the closely related guinea pig has retained control mechanisms for natural killer cells.

The authors next address the comparative gene expression of immune cells following lipopolysaccharide (LPS) injection in mice and naked mole-rats, both species activating similar pathways. However, interestingly, among the two neutrophil subgroups in the naked mole-rat, the naked mole-rat specific high lactotransferrin group increase in number and at the same time there was a convergence of the two groups, a result suggesting that although these two neutrophil subgroups exist with regard to gene transcription, they are in fact likely playing similar roles.

Overall, this is a really exciting paper that documents for the first time the immune system of the naked mole-rat, demonstrating key differences compared to other mammals. Importantly, this work is of a wide general interest and will provoke make future avenues of research e.g. how does the immune system of the naked mole-rat deal with a variety of challenges? This manuscript clearly establishes a platform for all future work to build upon.

- Indeed, this study was meant to serve as a springboard for future endeavors aiming to understand the connection between immune systems of non-model organisms and their unique health phenotypes.

Minor comments that should be addressed in a revised version:

- the authors give the age and sex of animals used, they should also state throughout as to whether or not the mice used were nulliparous, and whether or not the naked mole-rats were breeding or subordinate animals? This is stated for naked mole-rats used for blood smears on line 6 of the Materials and Methods, but not, as far as I can tell, for other parts.

- All mice used in our study were virgins and all naked mole-rats were non-breeders and therefore also virgins. We added these details to our Materials and Methods section, and now write on page 15: “The animals used for RNA sequencing in this study comprised 12 young C57BL/6 mice (eight to ten-weeks-old; six males, six females, all virgins) and 12 young NM-Rs (23-25-months-old; six males, six females, all non-breeders)”.

- naked mole-rats are naturally quite an inbred species, a situation likely pushed further through laboratory housing due to constraints of mixing and matching a species that is, as the authors note, both xenophobic and eusocial. Although such laboratory breeding would likely not explain the striking findings of this paper (e.g. absence of natural killer cells, two neutrophil subgroups and distinct LPS challenge response), it could perhaps influence how well the sequencing data obtained reflect the wild naked mole-rat immune cell transcriptome. Obviously, the same criticism could be aimed at any study using inbred mouse strains, but it would perhaps be helpful to know if animals throughout the study came from the same colony, or different colonies.

- The naked mole-rats used in this study came from ten different captive colonies. Although our captive population has been maintained in labs for ~30 years, given the long life spans of breeding females most of them are from the parental stock that is less than five generations out of the wild. As a result, it is unlikely that the main readout from our scRNA-seq data, which is transcriptionally defined clusters and hence cell types, would be different from those one would obtain using a random sample of wild caught naked mole-rats. In addition, we did verify that in each of our scRNA-seq datasets, each cluster was represented by all samples (e.g., see S1N and S1G Figs, for naked mole-rat and mouse spleens, respectively), eliminating the chance that our clusters are genotype-specific.

- what evidence do the authors have that markers used for different immune cell subgroups in the mouse are appropriate for use in the naked mole-rat, i.e. for the data in Figure 1A/B, Cxcr2 is used to identify neutrophils – how robust a marker is this in different species? I do not expect the authors to validate every marker, but a general discussion on this point would, I think, be helpful, especially for the more general reader.

- We thank the reviewer for raising this important point. Firstly, we realized that we should have included a more detailed description in our Materials and Methods section on how we assigned cell types to clusters. Briefly, we used sorted mouse immune-cell RNA-seq expression data from the ImmGen portal and intersected that with the cluster marker genes that we obtained to assign which ImmGen immune-cell type our marker genes most likely correspond to. This is now added to our Materials and Methods section – top of page 20, alongside an additional supplementary table (now S44 Table). This cell-type annotation approach was used for both species and we do admit that we lack sufficient knowledge in how conserved the various immune cell-type markers are between species, despite some work comparing human and mouse (PMID: 14978070 and bioRxiv: 574160). Notwithstanding, it is important to point out that we did not rely on only a single marker gene for any of the clusters (neither in the mouse nor in the naked mole-rat), as our main text might have wrongly suggested, and is now modified (Results section – page 5) to remove that notion. We hope that our revised version clarifies this important point.

**Reviewer #2 (Pontarotti Pierre, signed review):**

Review of the article Single-cell transcriptomics of the naked mole-rat reveals unexpected features of mammalian immunity" by Dr. Nimrod Daniel Rubinstein for PLOS Biology.

In summary the authors show that the naked -mole rat lost the NK cells and corresponding gene during the evolution of the Heterocephalus clade . This is an important finding that can help in the understanding (give more insight) of life longevity and cancer resistance mechanisms. In this regard the choice of the naked mole-rat model is appropriate.

My field is comparative and evolutionary genomic , therefore I have focused on this part of the article .

The in silico analyis (including comparative genomic ) is well described and robust and supports the authors conclusions .

- We thank the reviewer for his favorable review.

**Reviewer #3:**

The manuscript entitled “Single-cell transcriptomics of the naked mole-rat reveals unexpected features of mammalian immunity” describes a single cell RNA sequencing analysis of splenic cells in the naked mole rat compared to conventional mice. The interest of NMR is their apparent lower susceptibility to develop tumors.

- We would like to clarify that we chose to study the immune system of the naked mole-rat not only because of its resistance to tumor formation, but more importantly due to a broad range of remarkable health-associated phenotypes: prolonged lifespan, lack of physiological or molecular changes with age, and lack of age-associated exponential increase in risk of dying (all mentioned in the Abstract and Introduction). A well-honed immune system is thought to be critical for these phenotypes and therefore this is a more accurate description of the motivation for our study. Moreover, there is a dearth of knowledge regarding immune function in this model of exceptional biogerontological interest, hence this study is the first to comprehensively examine the naked mole-rat immune system.

The single cell RNA approach revealed that compared to mice the NMR has fewer B cells and more myeloid cells which skews the ratio lymphoid/myeloid cells that in the mouse is more than 2:1. Two additional features came out of the analysis, one was the apparent lack of NK cells and the other was the detection of a major population of LPS responsive neutrophils not seen in the mouse. This approach is of interest to immunologists and biologists in general. However, the data is solely descriptive and falls short of bringing concrete information on the function of the NMR immune system

- We agree with the reviewer that our paper includes several descriptive elements; however, as a first step in characterizing the immune system of a non-traditional model organism, descriptive text outlining the comparative cellular differences is essential and inevitable. It provides a framework for future studies addressing immune system function in the naked mole-rat and may additionally serve as a hypothesis generating tool. Therefore, we do not agree that it is a weak point in our work. Notwithstanding, some of our key findings are actually followed up with in-depth analyses – providing an evolutionary support for the absence of the naked mole-rat NK cells as well how its myeloid-biased immune system reacts to a bacterial-mimicking challenge. Clearly, additional functional studies focusing on specific immune cell subsets may further enhance our understanding of not only the immune system of the naked mole-rat, but also, whether some of these findings in non-model organisms may challenge our general understanding of mammalian immune function. Unfortunately, functional studies are still impeded by the lack of naked mole-rat specific tools and hence cannot be immediately executed.

Too much relevance is given here to establish a link between the lack of bona fide NK cells, the higher representation of myeloid cells and the low susceptibility to tumors in NMR. The major role of NK cells is a first line of defense to viral infection due to their capacity to detect cells with low or no MHCI expression modulated by many viruses. Tumor immune surveillance has also been described but other adaptive immune cells also play a role (see Smyth et al J. Exp. Med 2000 among others). In fact, recent strategies of cancer immune therapy focus on controlling check points within the adaptive immunity rather than stimulating the ILC compartment. That NMR do not have NK cells is not of major relevance to the discussion because other adaptive immune cells can also secrete cytokines, can take the protective role of NK cells and mechanisms other than immune response related might have evolved in the NMR (cell cycle arrest mechanisms, tumor suppressor genes such as P53 and

many other). More relevant in this context is the higher susceptibility of NMR to viral infection.

- Our Discussion previously included a sentence that may have over-emphasized an apparent paradox between lack of NK cells and cancer resistance in the naked mole-rat, implying that tumor immunosurveillance is the major role of NK cells (second sentence in the Discussion: “We find that the immune system of the NM-R is heavily reliant on innate myeloid lineage cells and contains a previously undescribed, transcriptionally distinctive cell subset. Paradoxical to its well documented cancer-resistance [2], we find that the NM-R lacks the innate lymphocyte considered crucial for cancer immunosurveillance – NK”). We have downplayed this in our Discussion and have emphasized that our findings, and namely the lack of NK cells, probably reflect relaxed viral selective pressures.

Also it should be clarified why there is a large population of erythroid cells found in the NMR and not in the mouse. Because RNA was obtained by total organ lysate this might indicate that in contrast to mouse the NMR erythroid cells have high amounts of RNA. Is the spleen a site of erythroid development in the NMR?

- We thank the reviewer for raising this point and our revised text now clarifies it (in the Materials and Methods section – bottom of page 20 and top of page 21) and with two additional supplementary figures – S11 and S12). Briefly, for obtaining single-immune-cell suspensions we applied the same protocols for mice and naked mole-rats, yet these were clearly designed and optimized for mice. Thus, it is possible that the efficiency of lysing erythroid cells is lower in the naked mole-rat due to biochemical differences in its cell-membrane components, relative to that of the mouse. That said, in addition to expression of adult hemoglobin genes, we also observed expression of embryonic and fetal hemoglobin genes in this population of cells, both in the spleen and in the circulation. In the spleen, we also observed that this population expresses a large number of genes (equivalent to that of other immune cell types), and therefore suspect that these may represent erythroid cells in various stages of maturation, and hence termed them so. Since our study is focused on the immune system we chose not to expand more on this population of cells and, unfortunately, we do not have an answer to whether the spleen is a site of erythroid development in the mature naked mole-rat.

Because the data is deposited in GEO are 48 Sup Tables needed?

- The data in GEO and in the supplementary tables are not identical. We deposited our raw sequence data (fastq files) as well as some processed data (read-mapping data – bam files) in GEO, as required. The supplementary tables mainly provide our cluster and cell type assignments as well as the cluster marker genes, all of which are outputs of our analyses. The purpose of having these supplementary tables is thus to enable easy access these data, which GEO does not typically store.

**Reviewer #4 (Jim Kaufman, signed review):**

This manuscript describes single cell RNAseq studies on spleen and blood cells of the naked mole rats in comparison with an inbred mouse strain, both unstimulated and after LPS treatment (as well as a big meta-analysis of gene copy number and a cell staining experiment). I am no expert on such single cell sequencing (although there is no escape from learning about it from many publications and seminars), but there is no question in my mind that this study is worthy of publication in a general high-impact journal of biology. Naked-mole rats are fascinating creatures with many unusual and unexplained characteristics, there is an enormous amount of work done in this paper which seems very well controlled, the results are extremely interesting and novel, the speculations will excite new functional experiments, and the manuscript is very carefully and well-written, so I would predict that this excellent paper will be cited in all studies

about this fascinating creature in several different scientific fields for years to come.

I have only two concerns for the authors to consider.

The first concern I believe that the authors should answer. They identify macrophages, red pulp macrophages and (Timeless-high) dendritic cells in the naked mole rat spleen, but only “antigen presenting cells” (APCs) in the mouse. I find this surprising, as the APCs of mice have been well-studied over many decades and so there are certainly RP and other macrophages as well several kinds of DCs in mouse spleen. I also note that the tSNE plots of the mouse cells show a lot of structure in the APC group, so it is well-possible that all these different kinds of cells are represented in that population. Perhaps I missed the key sentence in the text, or the explanation is buried in one of the many supplementary items (of which I looked at only a few), but I think that this point deserves explanation, if only a sentence.

- Macrophages and dendritic cells are indeed detected in the mouse data, yet at the converged stage of our clustering algorithm, whereas in the case of the naked mole-rat they are detected at the first iteration of the clustering algorithm. We now modified our text to point to that, writing in the Results section – top of page 5: “Myeloid lineage cells account for the remaining 8%, breaking down to 7% antigen presenting cells (e.g., marked by *Cd68*, *Ccl6*, and *C1qa*) (APCs, which resolve to macrophage and dendritic cell subsets at the converged clusters; S1E and S1F Figs)”.

The second concern is more general, but I believe could be dealt with in few sentences. The authors begin their manuscript by describing some of the unexpected features of the naked mole-rat, and their motivation for the study to examine the possibility that “the NM-R has evolved features of systemic immunity that contribute to these remarkable phenotypes.” By the end of the discussion, they have certainly shown that "the NM-R has evolved unusual features of systemic immunity”, but I didn’t feel that they drew conclusions from their data about the contribution "to the remarkable phenotypes". They include three other “histricomorphs” (Damaraland mole-rat, chinchilla and guinea pig) in their meta-analysis, but do not state clearly that their discoveries of cellular composition might be unique to the naked mole-rats or, alternatively, might be an evolutionary novelty for all the “histricomorphs” regardless of their social and ecological traits. Also, they correctly point out

that marine carnivores (and dogs, if I remember correctly) also have low levels of NK receptor genes, without exploring the implications (for instance, no or few NK cells apparently does not automatically mean more or less cancer, etc). There is a rich vein of interesting thoughts here with many further experiments to suggest, but I assume the authors wished to avoid a messy discussion and so compressed it all into the single sentence at the end of the discussion. As far as I am concerned, it is up to the authors, but they did leave me wishing for a bit more at the very end.

- Although our study was motivated by the well-known unique health-associated phenotypes of the naked mole-rat, which raises the question regarding the contribution of its immune system to its slow aging phenotype, we have avoided being too speculative in the Discussion. Reviewer #3 accurately reminded us that the major role of NK cells is to form a first line of defense against viral infections and although these cells have been described to play a role in cancer immunosurveillance in mice and humans, it is probably not of major of relevance to cancer resistance in the naked mole-rat. We therefore modified our Discussion accordingly, which now emphasizes that the lack of naked mole-rat NK cells as well as their myeloid bias, are probably a result of relaxed viral selective pressures. With regards to a more global interpretation of our results, and specifically the evolution of genes controlling NK cell function, our phyletic pattern analysis does have additional interesting findings. For example, NK cell receptor gene families have also eroded in some of the muroid rodents as well as in the dolphin species we analyzed, and there are weakly supported evidence suggesting that guinea pigs have a substantially higher cancer incidence (of multiple forms) in contrast to the other hystricomorphs we analyzed. However, since we strived to integrate our findings with strongly supported evidence, we chose not to expand these components in our Discussion.
